# A glimpse into the world of microRNAs and their putative roles in hard ticks

**DOI:** 10.3389/fcell.2024.1460705

**Published:** 2024-09-23

**Authors:** Brenda Leal-Galvan, Deepak Kumar, Shahid Karim, Perot Saelao, Donald B. Thomas, Adela Oliva Chavez

**Affiliations:** ^1^ Department of Entomology, Texas A&M University, College Station, TX, United States; ^2^ USDA-ARS Cattle Fever Tick Research Laboratory, Edinburg, TX, United States; ^3^ School of Biological, Environmental, and Earth Sciences, The University of Southern Mississippi, Hattiesburg, MS, United States; ^4^ USDA-ARS Veterinary Pest Research Unit, Kerrville, TX, United States; ^5^ Department of Entomology, University of Wisconsin—Madison, Madison, WI, United States

**Keywords:** extracellular vesicles, small RNA, tick feeding, RNA transport proteins, miRNA transport

## Abstract

Ticks are important blood feeding ectoparasites that transmit pathogens to wildlife, domestic animals, and humans. Hard ticks can feed for several days to weeks, nevertheless they often go undetected. This phenomenon can be explained by a tick’s ability to release analgesics, immunosuppressives, anticoagulants, and vasodilators within their saliva. Several studies have identified extracellular vesicles (EVs) as carriers of some of these effector molecules. Further, EVs, and their contents, enhance pathogen transmission, modulate immune responses, and delay wound healing. EVs are double lipid-membrane vesicles that transport intracellular cargo, including microRNAs (miRNAs) to recipient cells. miRNAs are involved in regulating gene expression post-transcriptionally. Interestingly, tick-derived miRNAs have been shown to enhance pathogen transmission and affect vital biological processes such as oviposition, blood digestion, and molting. miRNAs have been found within tick salivary EVs. This review focuses on current knowledge of miRNA loading into EVs and homologies reported in ticks. We also describe findings in tick miRNA profiles, including miRNAs packed within tick salivary EVs. Although no functional studies have been done to investigate the role of EV-derived miRNAs in tick feeding, we discuss the functional characterization of miRNAs in tick biology and pathogen transmission. Lastly, we propose the possible uses of tick miRNAs to develop management tools for tick control and to prevent pathogen transmission. The identification and functional characterization of conserved and tick-specific salivary miRNAs targeting important molecular and immunological pathways within the host could lead to the discovery of new therapeutics for the treatment of tick-borne and non-tick-borne human diseases.

## 1 Introduction

Ticks are important vectors of a wide range of disease-causing microorganisms that affect humans and other mammals (Anderson and Magnarelli, 2008; Nicholson et al., 2019). The incidence of tick-borne diseases has increased in recent years, raising public awareness about the importance of ticks. (Vayssier-Taussat et al., 2015; Lippi et al., 2021). In 2022, it was estimated that around 71 thousand people were infected with tick-borne pathogens, with reports of Babesiosis, Lyme Disease, Rocky Mountain Spotted Fever, and Anaplasmosis/Ehrlichiosis all rising in numbers (CDC, 2024b). The outcomes of these diseases range from self-resolving, in the case of Human Anaplasmosis, to fatality rates of 20%–30% for Rocky Mountain Spotted fever (Bakken and Dumler, 2015; Drexler et al., 2017; Bradshaw et al., 2020). Tick bites can also lead to the development of a delayed allergic hypersensitivity known as Alpha-Gal syndrome (AGS), which has also increased in incidence from 13,000 cases in 2017 to over 18,000 in 2021 (Thompson et al., 2023). According to the CDC, 110,000 cases of AGS have been reported between 2010 and 2022 (CDC, 2024a). In addition to their public health importance, ticks pose a global economic threat to livestock producers with $13.9–18.7 billion dollars loss annually (de Castro et al., 1997). Direct effects caused by tick feeding include cowhide damage, decreases in milk and meat yield, and anemia in the case of heavy infestations. Indirect effects from the transmission of tick-borne pathogens can result in animal mortality and abortions (Rajput et al., 2006).

Tick feeding and tick-borne pathogen transmission are facilitated by effector molecules within tick saliva that modulate immune responses at the bite site (Pham et al., 2021; Nuttall, 2023). Saliva assisted transmission has been reported during the inoculation of flaviviruses and bacteria in *in vivo* and *in vitro* experiments (Marchal et al., 2009; Hermance and Thangamani, 2015; Reynolds et al., 2023). Moreover, tick saliva can affect the clinical outcome of disease. In a recent study, Reynolds et al. (2023), showed that inoculation of the heartland virus along with salivary gland extracts (SGE) from *Amblyomma americanum,* the natural vector of this virus, enhanced virus persistence in the blood, had a significant effect on the reduction of lymphocytes, and led to higher liver inflammation in a murine model. Similar modulation of disease intensity and pathogen colonization has been observed using tick-salivary extracellular vesicles (EVs) (Oliva Chávez et al., 2021). However, large knowledge gaps concerning molecular factors involved in pathogen transmission and feeding hinder the development of efficient tick control tools and anti-tick transmission therapeutics and vaccines. Thus, understanding the molecular factors involved in pathogen transmission and feeding is essential to identify potential targets that can be used against ticks and tick-borne pathogens.

Although several groups have studied the properties of tick saliva, most studies have focused on profiling the sialome through proteomics, transcriptomics, or in the functional characterization of immunomodulatory or structural proteins (i.e., cement proteins), with little attention given to small RNAs (Madden et al., 2004; Perner et al., 2018; Martins et al., 2021). Small RNAs can be categorized as piwi-interacting RNA (piRNA), small interfering RNA (siRNA), microRNAs (miRNAs), and non-canonical small noncoding RNAs (sncRNAs), such as transfer RNAs (tRNAs), Y small RNAs, and others (Shi et al., 2022). From these small RNAs, miRNAs are the best studied due to their role in immune regulation and disease pathology. miRNAs are involved in post-transcriptional gene regulation to modulate homeostasis and respond to infection or environmental changes (Filipowicz et al., 2008; Hobert, 2008). In ticks, the role and characterization of miRNAs has just begun and there is limited understanding of their contribution in regulating tick biological processes. Even though it has not been shown experimentally, *in silico* analyses suggest that saliva-derived miRNAs may target transcripts of proteins involved in immune regulation in the host, potentially affecting their expression (Hackenberg et al., 2017). Recently, miRNAs were detected within tick salivary extracellular vesicles (EVs) secreted in the saliva of *Haemaphysalis longicornis* (Nawaz et al., 2020). The presence of miRNAs within the tick salivary EVs suggests that salivary miRNAs may act in the manipulation of host immune responses. Yet, most studies focused on salivary miRNAs, and EV-derived tick miRNAs, have been limited to profiling and putative function characterization. Therefore, the actual function of most of these miRNAS remains to be investigated. Similarly, how miRNAs are synthesized and the molecular mechanisms behind miRNAs loading into tick EVs are also largely unknown. This review discusses what is known about miRNA transport and packing into EVs in human cells as well as the tick miRNA profiles described to date. Lastly, we will finish with a discussion of potential avenues to exploit tick miRNAs.

## 2 Extracellular vesicles and their role in tick feeding

EVs have been studied in the context of cancer research and infectious diseases for their role in cell-cell communication (van Niel et al., 2018). EVs are categorized into three classes: exomeres (0–50 nm), exosomes (50–150 nm), and microvesicles (150 nm-1 µm) (Zhang et al., 2018). Exosomes are formed via the inward budding of multivesicular bodies (MVB), whereas microvesicles are produced by the outward budding and fission of the plasma membrane (Sedgwick and D'Souza-Schorey, 2018). How exomeres are formed is poorly understood (Zijlstra et al., 2018). Because the focus of this review is about the transport of miRNAs and their function, we will not describe the biogenesis of EVs further. For a comprehensive review of EV biogenesis and their cargo, refer to van Niel et al. (2018); Sedgwick and D'Souza-Schorey (2018).

EVs have the capability of transferring intracellular cargo from a donor cell to a recipient cell and are involved in interspecies interactions, such as plant-fungal, vector-pathogens, and host-parasite (Mu et al., 2014; Jurkoshek et al., 2016; Oliva Chávez et al., 2019; Freitas et al., 2020). In ticks, recent studies have shown that salivary derived EVs can manipulate skin host immunity and wound healing responses to promote successful feeding (Zhou et al., 2020; Oliva Chávez et al., 2021; Butler et al., 2023). Oliva Chávez et al. (2021) demonstrated that tick-derived EVs interact with immune cells and affect murine resident γδ T-cell migration and proliferation within the epidermis of mice. These cells belong to the dendritic epidermal T cells (DETCs). The knock-down of the EV biogenesis resulted in deficient tick feeding, which was rescued when the ticks fed on γδ T-cell deficient mice. Interestingly, exosomes isolated from the salivary glands and saliva of *Amblyomma maculatum* and *Ixodes scapularis* delayed wound healing during *in vitro* experiments with the human epidermal keratinocyte (HaCaT) cell line (Zhou et al., 2020). Upon injury, keratinocytes and γδ T-cells play critical roles in inflammation and wound healing responses. Moreover, γδ T-cells are essential for maintaining keratinocyte’s homeostasis in the epidermis (Chen et al., 2021). Even though it is currently unknown what specific molecules within EVs are involved in the suppression of inflammatory responses and the alteration of cellular homeostasis in the host, *in vivo* and *in vitro* experiments have shown that tick salivary EVs prevent wound healing and allow attached ticks to feed unabated (Zhou et al., 2020; Oliva Chávez et al., 2021). Further, the inoculation of tick salivary EVs along with the intracellular pathogen *Anaplasma phagocytophilum* enhanced its colonization and establishment in the skin (Oliva Chávez et al., 2021) and tick cell EVs assist in the translocation of Langat virus in *in vitro* models (Zhou et al., 2018). Thus, defining EV derived molecules that act in the regulation of host responses and identifying the molecular mechanisms for their packing within salivary EVs has important public health implications.

## 3 MicroRNA (miRNA): biogenesis and loading into EVs

**FIGURE 1:**
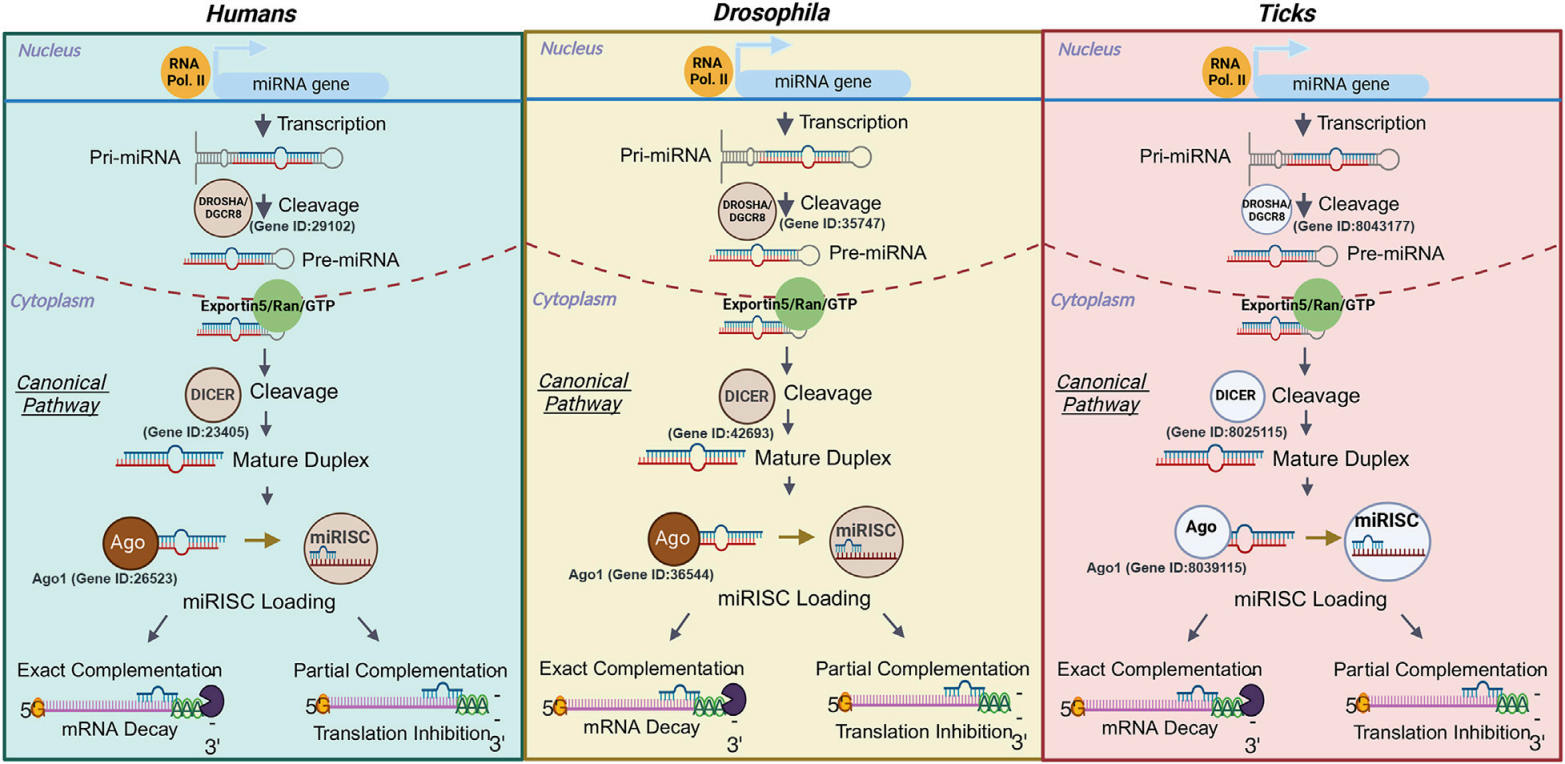
Comparison of the miRNA biogenesis canonical pathway in humans, Drosophila, and ticks. The transcription of a miRNA gene is completed by RNA polymerase II, which leads to the production of a miRNA. The primary miRNA is cleaved into a miRNA precursor by Drosha/DGCR8 and then exported out of the nucleus via Exportin 5/Ran/GTP. Next the precursor is cleaved by Dicer and forms a mature duplex that will be loaded into the miRNA Induced Silencing Complex by an Argonaut protein. The guide strand is then degraded, and the mature miRNA sequence will be translocated to its mRNA target. The exact complementation between the mRNA and miRNA leads to the degradation of the transcript, while the partial match represses translation. Ribonuclease III enzymes represented in dark or light brown have been functionally characterized in the species whereas their representation in white signifies that their role in silencing remains to be tested in members of this group. The ID of the gene encoding each of the ribonucleases has been added to each organism. In the case of ticks, the gene IDs from the *Ixodes scapularis* genome were used based on the results from Schnettler et al. (2014). Only Dicer 1 and Ago 1 IDs are provided as these proteins are involved in the miRNA silencing pathway. The IDs for Dicer 2 and Ago 2 in *I. scapularis* have been described in Schnettler et al. (2014). Images were created with BioRender.

miRNAs are small non-coding RNAs (snRNAs), ∼18–22 nucleotides in length, that are critical for regulating gene expression post-transcriptionally. miRNAs can inhibit protein translation or degrade target messenger RNA (mRNA) by binding to complementary sequences within the 3′ untranslated region (UTR) of mRNAs (Lu and Rothenberg, 2018). It is reported that miRNAs may regulate ∼60% of genes in humans and other mammals (Catalanotto et al., 2016; Shu et al., 2017). The biogenesis of miRNAs begins in the nucleus when a miRNA coding gene is transcribed into primary miRNAs (pri-miRNA, >1 kilobases) by RNA polymerase II (Lu and Rothenberg, 2018). Drosha and DiGeorge syndrome critical region 8 (DGCR or pasha), RNase III endoribonucleases, will cleave the pri-miRNAs into a miRNA precursor (pre-miRNA, ∼60–70 nt). The cleaving of pre-miRNAs by Drosha and DGCR leaves extended 5′phosphate and 3′ nucleotide overhangs (Kim et al., 2016; Sheng et al., 2018). The pre-miRNA is then transported to the cytoplasm by the Exportin 5 (XP05)/Ras-related nuclear protein GTPase (Ran-GTP) complex. The precursor extensions and loop structure is recognized and cleaved by DICER1, leaving a mature miRNA duplex known as the miRNA:miRNA* complex (miRNA = mature strand; miRNA* = guide strand (Yi et al., 2003; Kim et al., 2016). The mature duplex is transported into the RNA induced silencing complex (RISC) by the family of Argonaut (Ago) proteins, where the guide strand is degraded and only the mature strand is left (∼18–22 nucleotides). The Argonaut-miRNA duo is redirected to either repress or degrade the target mRNA (Sarshad et al., 2018; Iwakawa and Tomari, 2022). Conversely, how miRNAs are synthesized in ticks has yet to be defined. Studies assessing antiviral activity of the RNA interference (RNAi) pathway in ticks has shown that it is functional (Schnettler et al., 2014). Putative tick homologs of *Drosophila melanogaster* Ago-1 and Ago-2 have been identified in *Rhipicephalus* (*Boophilus) microplus* and *I. scapularis* (de la Fuente et al., 2007; Schnettler et al., 2014)*.* Additionally, Dicer-1 and Dicer-2 homologs have been identified in *I. scapularis* (Schnettler et al., 2014)*.* However, the involvement of these proteins in miRNA mediated interference has not been tested, but this canonical pathway is at least partially conserved in ticks as compared to *Drosophila* and humans (de la Fuente et al., 2007) (Figure 1).

**FIGURE 2:**
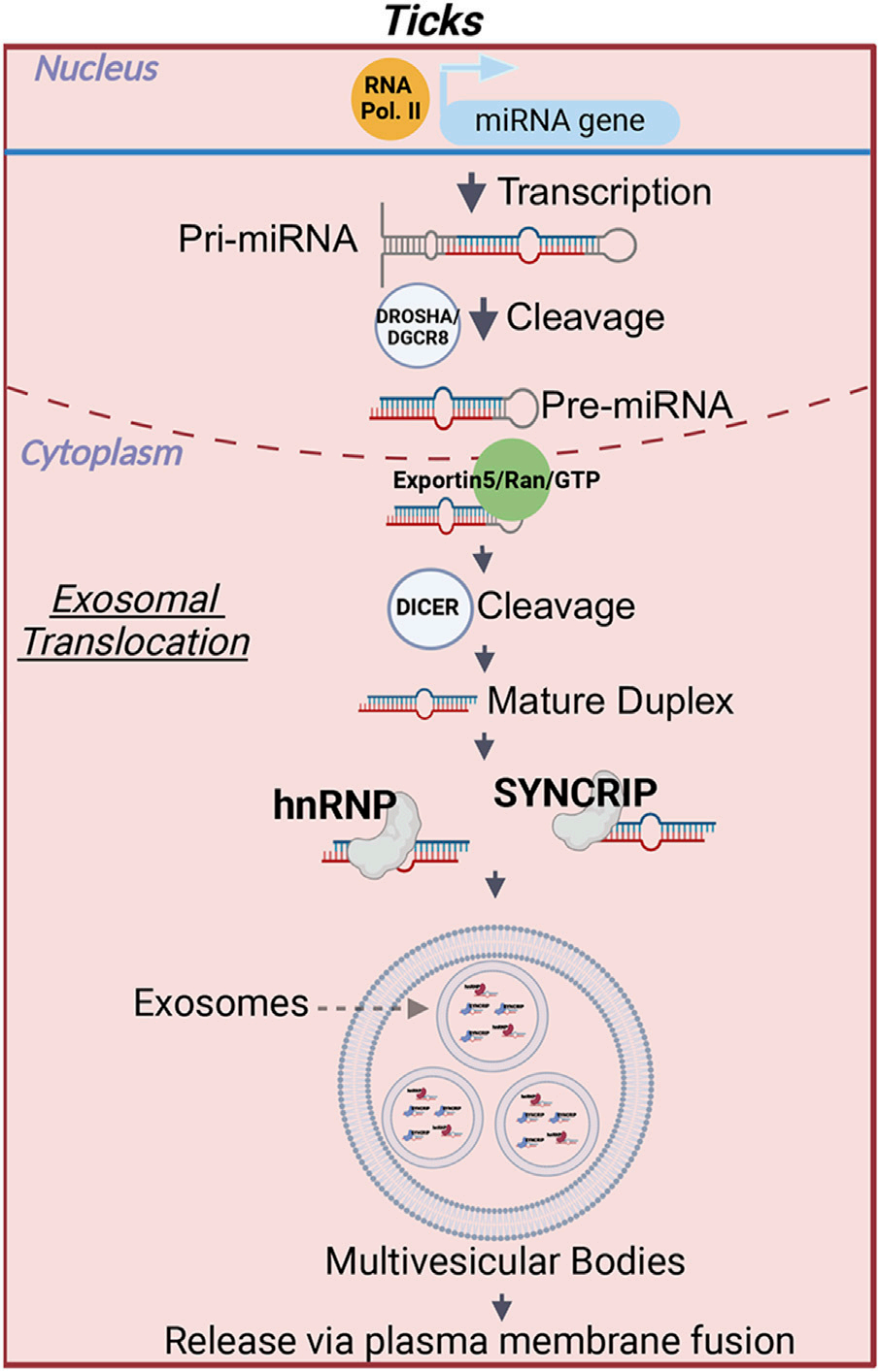
miRNA translocation into exosomes in ticks. After the cleavage of a precursor miRNA into a mature miRNA a motif in the seed sequence is recognized by RNA binding proteins, such as heterogenous nuclear ribonuclear protein A2B1 (hnRNPa2b1) and Synaptotagmin Binding Cytoplasmic RNA Interacting Protein (SYNCRIP). Bound miRNAs are loaded into exosomes. The exosomes will then be released when the multivesicular bodies fuse with the plasma membrane. The RNA binding proteins depicted in this figure have been shown to participate in miRNA translocation into exosomes in humans. Whether these proteins complete the same function in ticks remains to be determined. Image was created with BioRender.

Likewise, how miRNAs are packed within tick salivary exosomes for secretion is currently unknown. In human cells, the localization of miRNAs is determined by specific motifs that are recognized by transport proteins, which aid in their translocation into EVs (Lee et al., 2019; Groot and Lee, 2020; de Abreu et al., 2021). Villarroya-Beltri et al. (2013) demonstrated that when heterogeneous nuclear ribonucleoprotein A2B1 (hnRNPA2B1) undergoes SUMOylation, it facilitates the sorting of exosomal miRNAs through a GGAG/UGCA motif. Another member of the hnRNP family, Synaptotagmin-binding cytoplasmic RNA-interaction protein (SYNCRIP), also selectively sorted miRNAs containing a hEXOmotif GGCU (Santangelo et al., 2016; Hobor et al., 2018). Interestingly, these proteins do not appear to work in parallel, where SYNCRIP would not bind to miRNAs with the GGAG EXOmotif and hnRNPa2b1 would not bind with miRNAs containing the hEXOmotif GGCU (Santangelo et al., 2016). These findings suggest that RNA-binding proteins have a high level of specificity and each protein binds to unique EXOmotifs. A detailed review of the pathways involved in the miRNA sorting into exosomes in humans can be found in Xin et al. (2022). Interestingly, SYNCRIP is highly conserved between humans and arthropods, such as *D. melanogaster,* and domains important for RNA binding are present with high degree of similarity in these homologs (Hobor et al., 2018). This protein was recently shown to regulate the translation of mRNAs involved in synaptic function; however, this effect is due to direct binding of the protein to mRNAs (McDermott et al., 2014) rather than by the translocation of miRNAs into EVs. Likewise, a homolog of hnRNPa2b1, Hrb98DE*,* has been identified in *Drosophila* (Li et al., 2016)*.* Nonetheless, the involvement of these proteins in the transport of miRNAs into *Drosophila* exosomes remains to be determined. Similarly, RNA -binding protein Musashi (B7Q6Y7), a homolog of human hnRNPa2b1, has been detected by proteomic analysis of EVs from *I. scapularis* salivary gland *ex vivo* cultures (Oliva Chávez et al., 2021). The presence of B7Q6Y7 within salivary vesicles could indicate an involvement of this protein in the translocation of exosomal tick miRNAs, warranting further investigation (Figure 2).

In mammalian macrophages, Soluble N-ethylmaleimide-sensitive factor attachment protein receptors (SNARE) protein Syntaxin 5 also binds to miRNAs during the biosynthesis of exosomes and facilitates their export outside of the cell (Hom Choudhury et al., 2024). Human antigen R protein transfers miRNAs into a disordered domain in Syntaxin 5 that binds to miRNAs, such as let-7a and miR-122. Syntaxin 5 is then packed into exosomes along with these miRNAs, leading to their secretion outside the cell (Hom Choudhury et al., 2024). In ticks, SNARE proteins have been shown to assist in the secretion of tick salivary proteins (Karim et al., 2002; Karim et al., 2005) and tick derived exosomes (Oliva Chávez et al., 2021). Several SNARE proteins have been identified, including synaptobrevin-2, syntaxin-1A, syntaxin-2, SNAP-25, and Vamp33 (Karim et al., 2002; Oliva Chávez et al., 2021). By comparison, transport of miRNAs into microvesicles is less well defined, but experiments involving tumor cell derived microvesicles suggests that miRNA and pre-miRNA translocation is mediated by the interaction of the small GTPase ADP-ribosylation factor 6 (ARF6) and Exportin-5 (Muralidharan-Chari et al., 2009). Nonetheless, whether any of these mechanisms are involved in the trafficking of miRNAs into tick salivary vesicles remains to be determined. Corrado et al. (2021) and Groot and Lee (2020) expound on the mechanisms and details of miRNA transport and sorting.

## 4 miRNAs: profiles and putative roles

Because few studies have functionally characterized miRNAs in ticks, profiling can be used as a baseline to provide insight into their putative roles. A miRNA profile includes both novel, or organism specific, and conserved miRNAs. In the case of conserved miRNAs, their potential function can be inferred from their known role in the biology of other organisms or by looking at their expression profiles in specific organs, life-stages, tick species, during infection with tick-borne pathogens or entomopathogenic microbes, and in response to environmental stressors. For example, miRNA expression profiles of *Dermacentor silvarum* females exposed to four different temperatures, 8°C, 4°C, 0°C, −4°C, were compared during a 10-day treatment (Agwunobi et al., 2022). MiR-2a expression peaked at day 6 at 4°C and 8°C, whereas miR-279 was lowest at day 3 at all four temperatures. To understand their potential role in cold tolerance, these miRNAs were inhibited, altering the expression of its target genes, like glycogen phosphorylase (GPase) (Agwunobi et al., 2022). The inhibition of miR-2a and miR-279 significantly increased the mortality rates of ticks exposed to −22°C for 2 h in a thermostatic bath and then placed in a normal incubator to recover for 24 h, confirming that these miRNAs contribute to cold tolerance in *D. silvarum* (Agwunobi et al., 2022). A list of studies done to characterize the miRNA profiles within different ticks can be found in Table 1.

**TABLE 1:** List of studies describing microRNA profiles in hard ticks.

Species	Profile type	Tissue of interest	Function of interest	Characterized function	References
*Ixodes scapularis*	Vector-Pathogen	Salivary Glands	Saliva-assisted transmission	Pathogen Replication and Transmission	Hermance et al. (2019), Kumar et al. (2022)
*Hyalomma anatolicum*	Biological Processes	Whole Body	Large scale characterization	Development/Feeding	Luo et al. (2015), Luo et al. (2021)
*Haemaphysalis longicornis*	Biological Processes	Salivary Glands/Ovaries/Midgut	Feeding	Feeding/Digestion/Oviposition	Zhou et al. (2013), Malik et al. (2019a), Malik et al. (2019b)
*Dermacentor silvarum*	Environmental Influences	Whole Body	Cold Tolerance	Cold Tolerance	Agwunobi et al. (2022)
*Haemaphysalis longicornis*	Biological Processes	Extracellular Vesicles	Feeding	N/A	Nawaz et al. (2020)
*Haemaphysalis longicornis*	Wild And Cultured Populations	Whole Body	Gene Expression Regulation/Development	N/A	Luo et al. (2019), Liu et al. (2020b)
*Rhipicephalus sanguineus*	Biological Processes	Whole Body	Sex Differentiation	N/A	Shao et al. (2015)
*Rhipicephalus haemaphysaloides*	Biological Processes	Whole Body	Innate Immunity	N/A	Wang et al. (2015)
*Rhipicephalus (Boophilus) microplus*	Biological Processes	Whole Body	Development	N/A	Luo et al. (2022b)
*Rhipicephalus (Boophilus) microplus*	Conservation	Salivary Glands/Midgut/Ovaries	miRNA Conservation/Expression	N/A	Barrero et al. (2011)
*Ixodes ricinus*	Host-Manipulation	Salivary Glands	Feeding	N/A	Hackenberg et al. (2017)

^a^ The studies that were only profiled without function validation were marked with not available (N/A).

Even though miRNAs have been identified in various tick species under different conditions (Table 1), only one study has profiled miRNAs-EVs in *H. longicornis* saliva. Nawaz et al. (2020) identified 36 conserved and 34 novel miRNAs-EVs after 4 days of feeding. Among miRNAs of interest, miR-315 was the most abundant miRNA in salivary EVs. This miRNA has previously been reported in the saliva of several arthropod species, including *Aedes aegypti, Ae. albopictus,* and *Anopheles coluzzi* (Maharaj et al., 2015; Feng et al., 2018; Liu et al., 2020; Nawaz et al., 2020). Curiously, miR-315 was among the ten most abundant miRNAs in *H. longicornis* saliva yet was not found in high abundance in the salivary glands of fed *H. longicornis* adults (Malik et al., 2019a). This suggests that miR-315 is potentially bound to transport proteins or EVs and released into the saliva from the salivary glands during active feeding. Cellular depletion of EV secreted miRNAs is observed in macrophages (Hom Choudhury et al., 2024). Even though the function of miR-315 has not been characterized in any of the vector species listed above, it has been shown that miR-315 regulates the expression of Notum and Axin, two proteins involved in the repression of the Wnt/Wg signaling pathway in *Drosophila*. This repression of Wnt/Wg signaling leads to the development of wings from the notum in *Drosophila* (Silver et al., 2007)*.* In mammals, Wnt signaling affects the proliferation phase during skin wound repair by regulating keratinocyte migration, proliferation, and differentiation (Fathke et al., 2006; Zhang et al., 2015; Takaya et al., 2022). Activation of Wnt results in the phosphorylation of Dishevelled (Dsh) that binds Axin, leading to the accumulation of β-catenin in the cytoplasm (Kan et al., 2020). β-catenin is a protein involved in cell adhesion; however, it can act as a transcriptional regulator (Kim et al., 2017). The accumulation of β-catenin leads to its translocation to the nucleus where it activates keratinocyte proliferation (Bai et al., 2023). Yet, increased β-catenin in the nucleus of keratinocytes decreases wound healing by reducing keratinocyte migration (Stojadinovic et al., 2005).

Tick salivary exosomes from *I. scapularis* and *A. maculatum* delayed wound closure during *in vitro* tests using HaCaT cells, a human keratinocyte cell line. The molecular mechanism appears to be connected to the downregulation of CXCL12, which affects keratinocyte migration (Zhou et al., 2020). Activation of the canonical Wnt signaling pathway in stromal bone marrow cells reduces the expression and secretion of CXCL12 and decreases cell migration (Tamura et al., 2011). Given that miR-315 is found within the saliva of two different hematophagous arthropods and it is highly abundant in tick saliva and EVs (Malik et al., 2019a; Nawaz et al., 2020), it is highly likely this miRNA could target Axin in keratinocytes upregulating the Wnt signaling pathway and decreasing CXCL12 expression. Interestingly, target prediction analysis of genes potentially affected by mir-1_38, mir-252_6, and mir-252_2, miRNAs found within the saliva of *Ornithodoros moubata* and *O. erraticus,* showed an enrichment of genes involved in the Wnt signaling pathway and angiogenesis. Gene targets of interest included Chondromodulin (CNMD), TRAF interacting protein with forkhead associated domain (TIF), and Growth hormone secretagogue receptor (GHSR), which are involved in angiogenesis and immune signaling (Cano-Argüelles et al., 2023). Still, whether tick miRNAs and EV-miRNAs are involved in the repression of wound healing remains to be determined.

### 4.1 Conserved and tick-specific microRNAs

The miRNA seed region, the first 6-8 nucleotides of the mature sequence, or the flanking sequences of the precursor can be highly conserved across various arthropod taxa (Etebari et al., 2015; He et al., 2017; Qu et al., 2017). In some cases, these conserved miRNAs might maintain similar functions or play multiple roles, even within the same organism (Table 2). For instance, Let-7 is a highly conserved miRNA, that clusters with miR-100/-125, and plays a critical part in development, feeding, reproduction and pathogen replication in *Ae. Albopictus*, *Hyalomma asiaticum*, and *Drosophila* (Sokol et al., 2008; Gu et al., 2013; Wu et al., 2019). In *H. anatolicum* females, miR-1 was consistently expressed throughout each life stage and targeted the gene encoding Heat shock protein 60 (Hsp60) (Luo et al., 2021). When miR-1 was inhibited, the ticks displayed obvious deformities during the later developmental stages and slower engorgement time. Nonetheless, a compensatory effect by close miRNA family members is sometimes observed when another member is inhibited. This compensatory effect is observed by the upregulation of a miRNA that targets the same mRNA as the family member that has been knocked down. These miRNAs are often transcribed within the same miRNA encoding gene but may differ by one nucleotide (Pidíkova et al., 2020; Donnelly et al., 2022). This is a common phenomenon reported in invertebrates and vertebrates (Abbott et al., 2005; Trümbach and Prakash, 2015). This compensatory effect might explain the upregulation of miR-5, a miRNA closely related to miR-1 that also recognizes the same target site in the Hsp60 mRNA (Luo et al., 2021).

**TABLE 2:** Conserved microRNA and their putative or validated roles in hard ticks, mosquitoes, and *Drosophila*.

Species	Role	Sequence	References
miR-1			
*Hyalomma anatolicum*	∆ Development/Feeding	N/A	Luo et al. (2021)
*Drosophila*	∆ Development	CCA​UGC​UUC​CUU​GCA​UUC​AAU​A	Sokol and Ambros (2005)
Let-7			
*Hyalomma asiaticum*	Molting	N/A	Wu et al. (2019)
*Anopheles gambiae*	Immunity	UGA​GGU​AGU​UGG​UUG​UAU​AGU	Fu et al. (2020)
*Drosophila*	∆ Development	UGA​GGU​AGU​AGG​UUG​UAU​AGU	Sempere et al. (2002), Sokol et al. (2008)
miR-375			
*Haemaphysalis longicornis*	*Oviposition and hatchability	N/A	Malik et al. (2019a)
*Aedes aegypti/*Aag 2 cell line	∆Development/pathogen transmission	UUU​GUU​CGU​UUG​GCU​CGA​GUU​A	Hussain et al. (2013)
*Drosophila*	Olfactory memory	ACU​UGG​GCC​AAG​GGA​AUG​CAA​ACU	Rahman et al. (2020)
miR-275			
*Haemaphysalis longicornis*	Reproductive development/Blood digestion	N/A	Hao et al. (2017)
*Aedes aegypti*	Egg development/blood digestion	CGC​GCU​AAG​CAG​GAA​CCG​AGA​C	Zhao et al. (2017), He et al. (2021)
*Drosophila*	Male spermatid maturation	CGC​GCU​AAU​CAG​UGA​CCG​GGG​CU	Eun et al. (2013)
miR-71			
*Ixodes scapularis*	*Pathogen replication	UGA​AAG​ACA​UGG​GUA​GUG​AGA​UG	Kumar et al. (2022)
*Culex pipiens pallens*	Pesticide resistance	N/A	Guo et al. (2017)
*Drosophila*	∆ Development	N/A	Leaman et al. (2005)

**FIGURE 3:**
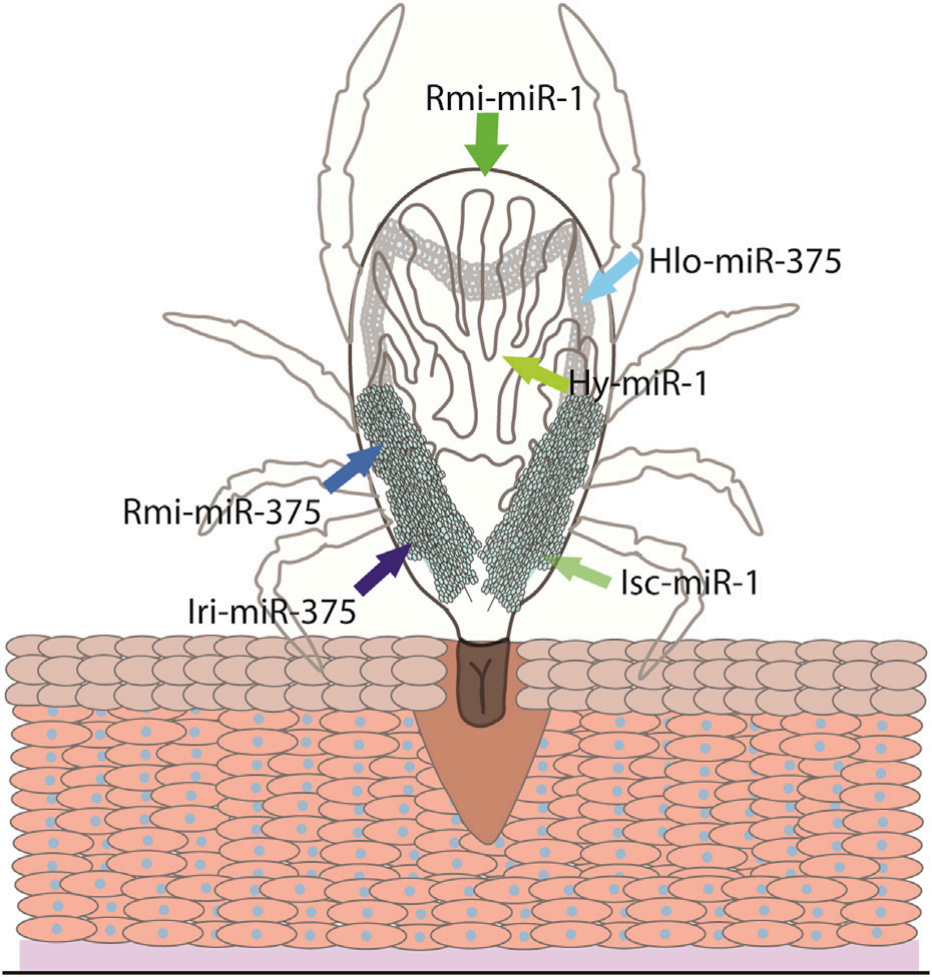
The conserved miRNA-1 and miRNA-375 and their site of expression. The site of expression of miRNA-1 (green arrows) and miRNA-375 (blue arrows) have been investigated in *Ixodes scapularis* (Isc), *Rhipicephalus microplus* (Rmi), *Hyalomma Anatolicum* (Hy), *Haemaphysalis longicornis* (Hlo), and *Ixodes ricinus* (Iri).

Some conserved miRNAs are regulators of biological processes, such as feeding or oviposition among hard ticks. A conserved miRNA, miR-375, is a highly expressed miRNA in the salivary glands, saliva and salivary EVs of *H. longicornis* and in the saliva of *I. ricinus* (Hackenberg et al., 2017; Malik et al., 2019a; Nawaz et al., 2020). Oddly, despite lower expressions of this miRNA in the ovaries and midgut, when miR-375 was inhibited in *H. longicornis*, feeding was not affected, instead resulting in a decrease of egg laying and eclosion (Malik et al., 2019a) (Figure 3). This may indicate miR-375 could directly or indirectly affect oviposition. MiR-375 is a prime example of a miRNA that is conserved across different taxa and has a diverse range of targets. For example, studies have shown that miR-375 can be involved in immunity, insulin secretion, and development in humans, *A. aegypti,* and *Culex pipiens* (Hussain et al., 2013; Jafarian et al., 2015; Meuti et al., 2018). A recent study using *Ixodes ricinus* showed that silencing an insulin receptor and two signaling proteins involved in the insulin signaling pathway, AKT and TOR, resulted in reduced weight during feeding and lower oviposition (Kozelková et al., 2021). Thus, miR-375 may affect several biological processes within ticks by regulating the insulin signaling pathway.

Unlike conserved miRNAs, novel miRNAs are species specific and share no homology with miRNAs identified in other organisms. In the case of tick specific miRNAs, these miRNAs might regulate biological processes unique to ticks. For instance, in *H. asiaticum* females, tick-specific miRNA-17 (nov-miR-17) was shown to affect blood-feeding by repressing the expression of the gene coding transforming growth factor-β (TGF-β)-activated kinase 1 binding protein (TAB2) and TGF-beta receptor type I (TβR-I) (Luo et al., 2022a). Injection of ticks with nov-miR-17 agomirs, synthetic mimics of endogenous miRNAs, led to the downregulation of TAB2 and TβR-I, resulting in reduced weight after engorgement. By contrast, antagomir treatment, chemically synthesized oligonucleotides complementary to miRNAs that leads to their silencing (Krützfeldt et al., 2005), led to enhanced expression of TAB2 and TβR-I and increased bloodmeal weight (Luo et al., 2022a). Although the exact molecular mechanism behind TAB2 and TβR-I regulation of tick feeding remains to be further described, TAB2 is a binding partner of the transforming growth factor-β activated kinase 1 (TAK1), part of the Immune Deficiency (IMD) signaling pathway in ticks (Oliva Chávez et al., 2017). Interestingly, the IMD pathway activates the shedding of epithelial cells in *Drosophila* midguts upon gram-negative bacteria infection (Zhai et al., 2018). Tick digestion occurs intracellularly within midgut epithelial cells that detach from the base membrane during gut cell remodeling (Sojka et al., 2013) and it is well documented that the microbiota of tick midguts is altered upon blood-feeding (Boulanger et al., 2023). Changes in microbiota during blood feeding could lead to the activation of the IMD pathway that may be further regulated by miRNA-17, hence affecting midgut epithelium shedding.

A tick-specific miRNA that appears to be involved in the regulation of development is novel miR-2 (Hlo-miR-2). This miRNA was found to be highly abundant in unfed ticks during all life stages, except in the eggs (Liu et al., 2020). This miRNA is highly expressed in epidermal and midgut tissues. Its target is the cuticular protein 1 (CPR1), which belongs to the most abundant family of insect cuticular proteins that bind to chitin and is present in most arthropods (Cornman et al., 2008). When Hlo-miR-2 was overexpressed, the expression of CPR1 was significantly reduced, resulting in delayed molting and obvious morphological defects in molted nymphs. Blocking miR-2 with antagomirs, on the other hand, reduced molting time in nymphs (Liu et al., 2020), therefore, demonstrating the importance of tick-specific miRNAs in regulating tick biological processes.

## 5 Arthropod miRNAs in host manipulation

During active feeding, ticks and other parasitic arthropods release salivary immunomodulatory compounds into the skin bite site to counteract the host immune system (Šimo et al., 2017; Perner et al., 2018). There is no experimental demonstration of hematophagous arthropods utilizing their salivary miRNAs to manipulate host responses at the bite site. However, *in vitro* experiments transfecting VeroE6 cells, an epithelial cell line derived from African green monkey kidney, with inhibitors of 9 *I. scapularis* miRNAs upregulated during Powassan virus (POWV) infection, affected the replication of the virus in the mammalian cells. Transfection of VeroE6 cells with isc-miR-124 and isc-miR-184 inhibitors resulted in decreased viral loads compared to mock transfected cells. By contrast, 6 isc-miRNA inhibitors, including inhibitors against the tick-specific isc-nDS630914_20990, isc-nDS625977_65388, and isc-nDS752087_3745 inhibitors, led to increased viral titers (Hermance et al., 2019). Although, not tested by the authors, it is possible that the isc-miRNA inhibitors interacted with miRNAs in the mammalian host cells, mimicking the potential effect of the tick miRNAs. isc-miRNAs might target the same mRNAs as their mammalian homologs, leading to the repression of mammalian proteins. This phenomenon has not been reported during feeding of hematophagous arthropods in mammalian hosts. However, arthropods infesting plants can regulate plant genes by secreting miRNAs in their saliva. The rice planthopper (*Nilaparvata lugens*) secretes miR-7–5p, which represses the bZIP transcription factor 43 and plant defenses. This miRNA is injected into rice plants during feeding and its inhibition reduced phloem sap ingestion (Zhang et al., 2024). Thus, arthropod salivary miRNAs are capable of cross-kingdom RNAi interference; yet, whether hematophagous arthropods inject miRNAs that can reduce the expression of host genes remains to be determined.

*In silico* studies on hematophagous arthropods suggest that tick salivary miRNAs target host genes. Putative target analysis of miRNAs overexpressed in *I. ricinus* saliva, when compared to salivary glands, showed that saliva overexpressed miRNAs target host physiological responses. Four miRNAs overexpressed in *I. ricinus* saliva, miR-8–3p, miR-279a–3p, miR-bantam-3, and miR-317-3p potentially regulate mitogen-activated protein kinases (MAPK), like MAPK10 (Hackenberg et al., 2017), a JNK pathway signaling protein previously shown to be regulated by miRNAs and modulate wound healing in mice (Liu et al., 2023). Surprisingly, other mRNAs potentially targeted by these miRNAs included transcripts encoding proteins involved in the mTOR signaling pathway (Hackenberg et al., 2017). A potential similar effect has been predicted in other hematophagous arthropods. miRNAs released within the saliva of *A. coluzzi* can mimic human endogenous miRNAs to potentially manipulate their host (Arcà et al., 2019). Target prediction analysis of the eight most abundant miRNAs in *A. coluzzi* saliva identified potential mammalian targets like rapamycin (mTOR), Phosphatidylinositol −4,5-bisphosate 3-kinase catalytic subunit delta (PIK3CD), Fc fragment of IgG receptor IIIb (FCGR3B), and several other proteins involved in T cell regulation and inflammation. Other potential targets for *A. coluzzi* salivary miRNAs included the transcriptional factors NF-κB, NFAT1, and IRF4, chemokines (such as CCL2 and CCL8), the signaling factor MyD88, and other immune related genes. Interestingly, certain miRNAs identified in mosquito saliva have also been detected in the saliva of *I. ricinus,* including miR-276-3p, miR-263a–5p, miR-100-5p, and several others (Arcà et al., 2019). Given that salivary miRNAs and their targets are conserved among different arthropod species, it is likely that distinct tick species target similar pathways in different hosts, although the miRNA that is utilized for the regulation of a pathway may differ depending on the tick species. Even though no experimental data has been provided to validate this hypothesis; nonetheless, these studies provide insight into how ticks might potentially use miRNA to regulate host responses and facilitate their uninterrupted feeding.

## 6 Potential application avenues for tick salivary miRNA

Due to the importance of miRNAs in gene regulation, development, and host-arthropod interactions, miRNAs are being explored as novel pest management tools against herbivorous insects. Insect feeding or development can be disturbed by delivering miRNAs to disrupt important biological processes in a strategy known as trans-kingdom RNA interference (TK-RNAi) or through the delivery of endogenous miRNAs *via* viral-vectors, nanoparticles, or exosomes (Bordoloi and Agarwala, 2021; Zhang et al., 2021). In TK-RNAi, artificial miRNAs (amiRNAs) can be introduced into target pests when insects ingest bacteria modified to express endogenous miRNA precursors (Zhang et al., 2021), a technology that could be exploited to reduce blood feeding in livestock. Ticks are pool-feeders that intake blood accumulated at the bite site after cutting small blood vessels in the skin with their chelicerae. Therefore, bacteria from the skin are likely to be ingested during the bloodmeal and modify the microbiome present in ticks. In fact, Boulanger et al. (2023) showed that at least three different genera of bacteria found in the skin of mice, originally detected in low proportions in unfed ticks, increased in prevalence after feeding. Bacteria present in the skin microbiome of animals, like livestock, and shown to be ingested by ticks during feeding, could be genetically modified to express precursors from tick miRNAs that disrupt crucial biological processes, like oviposition or molting. These genetically modified bacteria could then be inoculated into the skin of these animals, potentially reducing tick populations by decreasing egg numbers or lowering ability to molt to their next life stage when ingested with the bloodmeal. Likewise, engineered exosomes could be used to distribute antagomirs targeting miRNAs involved in the immune modulation of the host to reduce tick feeding and pathogen transmission. These vesicles carrying antagomirs could also be used in conjunction with vaccination programs in high infestation areas to increase immune responses in livestock.

On the other hand, tick derived miRNAs could be explored as potential therapeutics. Ticks have evolved salivary compounds to modulate host processes. These compounds can be exploited in the discovery of novel therapeutics for humans and other vertebrates that can be used systemically for the treatment of non-tick related illnesses (Aounallah et al., 2020). One example is Amblyomin-X, a 13.5 kDa protein encoded within the *Amblyomma cajennense* genome that acts as a Factor Xa (FXa) inhibitor (Batista et al., 2010). This protein reduced viability of tumor cells by inducing ER stress (Morais et al., 2016) and can decrease melanoma size after intra-tumor injection in horses (Lichtenstein et al., 2020). Amblyomin-X is listed as an anti-tumor drug on the NIH National Cancer Institute website (NIH, 2024). Though experimental evidence displaying the effect of miRNAs in host gene regulation is still lacking, the conservation of miRNAs in blood-feeding arthropods (Arcà et al., 2019) and their potential targeting and manipulating host immune, inflammatory, and wound related pathways (Hackenberg et al., 2017; Cano-Argüelles et al., 2023) makes them interesting targets for the development of novel therapeutics for tick-borne and other human diseases. In fact, delivery of miRNAs within exosomes is being explored as an avenue to speed wound closure (Liu et al., 2023), increase the susceptibility of cancer cells to treatment (Lou et al., 2020), reduce aorta inflammation to increase survival during aneurysm (Hu et al., 2022), and other disorders affecting humans. Remarkably, some of the proteins and pathways being focused on for the development of exosome mediated therapeutics in humans are putative targets of tick miRNAs, including mTOR and MAPK10 (Lou et al., 2020; Liu et al., 2023). Thus, profiling of salivary tick miRNAs and EV-derived miRNAs will not only shed light on the biology of tick feeding and their interaction with their host but may also help identify unexplored avenues for the development of human and animal treatments against cancer and other illnesses.

Similarly, the study of other small RNAs within tick saliva and EVs may reveal important information about tick biology, modulation of immune responses in the host, and disease pathogenesis. Paradigm shifting studies have shown that glycosylated (sialylated and fucosylated) small RNAs are found exposed on the outside of the plasma membrane of mammalian cells, particularly Y RNAs, small nuclear RNAs (snuRNAs), small nucleolar RNAs (snoRNAs), and tRNAs (Flynn et al., 2021); populations of small RNAs known to be present within EVs (Dellar et al., 2022). These glycoRNAs play a role in neutrophil recruitment and migration through the endothelium (Zhang et al., 2024) and could potentially enter the secretory system displayed in EV membranes (Nachtergaele and Krishnan, 2021; Dellar et al., 2022). Although Y RNAs have not been reported within tick salivary EVs, tRNAs and snoRNAs have been identified in *H. longicornis* salivary EVs (Nawaz et al., 2020) and small RNAs between 20–150 nucleotides have been observed *I. scapularis* salivary EVs (Leal-Galvan et al., 2022). Glycosylation, sialylation, and fucosylation are associated with the development of AGS (Karim et al., 2023) and the entry of *A. phagocytophilum,* the causative agent of Human Granulocytic Anaplasmosis (HGA), into human, murine, and tick cells (Carlyon et al., 2003; Pedra et al., 2010; Ojogun et al., 2012). Curiously, *A. phagocytophilum* hijacks the secretory vacuoles where EVs are formed (Read et al., 2022), and it is known to increase EV secretion in tick cells (Oliva Chávez et al., 2021). Whether small RNAs from ticks are modified remains unknown, but its potential implication in AGS and other tick-borne disease pathologies warrants further exploration.

## 7 Conclusion and concluding remarks

Because of their role in post-transcriptional gene regulation, miRNAs have been explored in various prospective applications ranging from disease diagnosis in humans (Ghamlouche et al., 2023) and in domestic animals (Winter et al., 2021) to the development of novel pest control in plants (Bordoloi and Agarwala, 2021; Zhang et al., 2021). Comparatively, our grasp of the capabilities of small non-coding RNA in regulating tick innate immunity, viral replication, and other biological processes has only begun. Nevertheless, progress in investigating the role of miRNAs in tick biology is held back by the difficulties of working with non-model organisms. The challenges when investigating miRNAs in hard ticks include the lack of well-annotated genomes (Xiong et al., 2020), the compensatory role of miRNA family members that may mask the results from functional analysis (Donnelly et al., 2022), non-coding RNA cross-talk (Tan et al., 2015), shortage of bioinformatic software tailored to non-model organisms (Bortolomeazzi et al., 2019), and presence of unidentified vectored pathogens that may alter miRNA profiles or results.

Several current efforts are trying to increase the number and quality of tick genomes available for bioinformatic analysis (Jia et al., 2020; Yu et al., 2022; De et al., 2023; Nuss et al., 2023). For example, initiatives within the USDA Agricultural Research Services, like the AgPest 100 and Veterinary Pest Genetics Research Unit, are developing high quality genome assemblies via PacBio HiFi reads and HiC contact mapping (I5K, 2021). However, investigators must still be mindful of the BUSCO score that signifies the completeness of the assembly and whether it is assembled at the chromosome or scaffold level (Yandell and Ence, 2012; Seppey et al., 2019). Currently, only the *I. scapularis* genome (GCA_031841145.1), *Rhipicephalus sanguineus* (GCA_013339695.2), *R. B. microplus* (GCA_013339725.1), *D. silvarum* (GCA_013339745.2) and *H. longicornis* (GCA_013339765.2) are annotated to the chromosome level with some unplaced scaffolds (Jia et al., 2020; Nuss et al., 2023). However, some tick genomes may not include the annotation of miRNAs, thus, software used to identify miRNAs would only provide the chromosome or genomic coordinates. Tools like miRDeep2 (Friedländer et al., 2008; Friedländer et al., 2012) or sRNAtoolbox (Aparicio-Puerta et al., 2022) identify sequences based on the miRNA biogenesis pathway. Still, these tools might detect false positives. Therefore, stringent filtering protocols to remove the false positives or low-quality miRNAs are needed; especially when reporting novel miRNAs. These improvements in analysis during profiling, *in silico* identification of miRNA targets, and *in vitro* and *in vivo* validation of miRNA function particularly in salivary glands and tick saliva will lead us to identify tick specific miRNAs. This information could potentially be used for the development of novel therapeutics for treatment in human related illnesses and the identification of targets that decrease the immunomodulation caused by ticks. Likewise, the recent finding that tick salivary EVs transport miRNAs (Nawaz et al., 2020) has opened a new array of possible miRNA delivery mechanisms utilized for tick control.
